# Experimental and Modeling Study of Drug Release from HPMC-Based Erodible Oral Thin Films

**DOI:** 10.3390/pharmaceutics10040222

**Published:** 2018-11-09

**Authors:** Alessandra Adrover, Gabriele Varani, Patrizia Paolicelli, Stefania Petralito, Laura Di Muzio, Maria Antonietta Casadei, Ingunn Tho

**Affiliations:** 1 Dipartimento di Ingegneria Chimica, Materiali e Ambiente, Sapienza Universitá di Roma, Via Eudossiana 18, 00184 Rome, Italy; gabriele.varani@uniroma1.it; 2 Dipartimento di Chimica e Tecnologie del Farmaco, Sapienza Universitá di Roma, Piazzale Aldo Moro 5, 00185 Rome, Italy; patrizia.paolicelli@uniroma1.it (P.P.); stefania.petralito@uniroma1.it (S.P.); laura.dimuzio@uniroma1.it (L.D.M.); mariaantonietta.casadei@uniroma1.it (M.A.C.); 3 Section of Pharmaceutics and Social Pharmacy, Department of Pharmacy, Faculty of Mathematics and Natural Sciences, University of Oslo, 0316 Oslo, Norway; ingunn.tho@farmasi.uio.no

**Keywords:** erodible thin films, HPMC, cyclodextrins, furosemide, Franz cell, USP II, millifluidic flow-through device, erosion

## Abstract

In this work hydroxypropyl methylcellulose (HPMC) fast-dissolving thin films for oral administration are investigated. Furosemide (Class IV of the Biopharmaceutical Classification System) has been used as a model drug for in vitro release tests using three different set-ups: the Franz cell, the millifluidic flow-through device, and the paddle type dissolution apparatus (USP II). In order to enable drug incorporation within HPMC films, a multifunctional excipient, hydroxypropyl-β-cyclodextrin (HP-β-CD) has been included in the formulation, and the influence of HP-β-CD on film swelling, erosion, and release properties has been investigated. Mathematical models capable of describing the swelling and release processes from HPMC erodible thin films in different apparatuses have been developed. In particular, we propose a new model for the description of drug transport and release in a Franz cell that accounts for the effect of the unavoidable imperfect mixing of the receptor chamber.

## 1. Introduction

Most oral dosage forms are designed to be swallowed as a single unit delivering a precise dose of the drug. Recent data suggest that dosage forms are far from optimal for pediatric or geriatric patients who have difficulties, or are unable to swallow tablet or capsules [1]. The exact incidence of patients with impaired ability to swallow (i.e., dysphagia) is not exactly known [2]. It is a known complication of different pathological conditions, such as stroke, dementia, and chronic obstructive pulmonary disease (COPD), and it is often observed in patients suffering from progressive neurological diseases (e.g., Parkinson’s disease), and head and neck cancers. Indeed, it is estimated that 20% of the population have psychological or physiological impairments that prevent them from swallowing tablets or capsules. Most of these patients are unwilling to take these formulations due to fear of choking, which results in a high incidence of non-compliance and failure of pharmacological therapies.

The use of alternative oral dosage forms should be carefully considered. Often patients with dysphagia or their care providers may alter formulations of medication to facilitate swallowing. Medication administration errors have been identified three times more frequent in patients with dysphagia [3]. The administration of crushed tablets and the contents of opened capsules exposes the patient to the unpleasant taste of the drug and potentially reduced efficacy of the medication. To overcome these issues, there are new and improved methods to deliver drugs to the oral cavity [4]. One such novel approach is represented by fast dissolving oral thin films (OTFs) able to increase consumer acceptance by rapid disintegration, self-administration without the need for drinking or chewing [5,6].

OTFs, or oral strips, are a stamp-size dosage form that employs a water-soluble polymer, which allows the dosage form to quickly hydrate by saliva, adhere to mucosa, rapidly disintegrate, and dissolve in the mouth, thus releasing the drug for both oromucosal and intestinal absorption. These aspects make them an alternative and more convenient solid dosage form, especially for patients with swallowing problems. In fact, OTFs can solve the issues related to tablets and capsules, while guaranteeing the benefits related to solid-dosage forms and, for these reasons, they are gaining increasing interest.

Hydroxypropyl methylcellulose, also named hypromellose (HPMC), is a water-soluble polymer commonly used as film-forming material. Within this study OTFs for furosemide (FUR) delivery were produced by solvent casting technique, using hydroxypropyl methylcellulose (HPMC) as matrix-forming. Data on solubility, oral absorption, and permeability classify FUR into Class IV of the Biopharmaceutical Classification System [7,8].

In order to enable drug incorporation within HPMC films, a multifunctional excipient was included in the formulation. Hydroxypropyl-beta-cyclodextrin (HP-β-CD) was used because it may fulfill several different roles, i.e., it may increase the loading efficiency of the OTFs while avoiding drug recrystallization [9], and it helps the taste-masking and improves drug absorption [10,11,12].

Work is mainly focused on the study, analysis, and modeling of the in vitro release of furosemide from HPMC-based OTFs, with and without the inclusion of HP-β-CD.

Three different apparatuses, namely the Franz cell, the millifluidic flow-through device [13,14,15], and the USP II paddle type dissolution apparatus, were used and the results compared.

Mathematical models capable of describing the release process from erodible OTFs in different apparatuses have been developed. In particular we propose a new mathematical approach to describe drug transport and release in a Franz cell, by removing a standard assumption, namely that of perfect mixing in the accepting chamber.

## 2. Materials and Methods

### 2.1. Materials

Hypromellose 5 (HPMC5, 5 mPas at 1% *w*/*w* and 25 ${}^{\circ }$C) and glycerol were obtained from NMD, Oslo, Norway. Potassium phosphate monobasic (KH${}_{2}$PO${}_{4}$), sodium phosphate dibasic dihydrate (Na${}_{2}$HPO${}_{4}$), potassium permanganate (KMnO${}_{4}$), and sodium chloride (NaCl) used for the preparation of simulated saliva were purchased from Merck, Darmstadt, Germany. Methanol (MeOH) was from VWR BHD Prolabo, Singapore. 2-Hydroxypropyl-β-cyclodextrins (HP-β-CD), Cavasol W7 HP Pharma, degree of substitution 0.6–0.9 mol per anhydroglucose unit, M${}_{w}$≃ 1396 g/mol, was from ISP, Koln, Germany. Furosemide and ethanol were purchased from Sigma Aldrich, Steinheim, Germany.

MQ water produced by dispenser Milli-Q Integral 3 Water Purification System, Merck Millipore, Billerica, MA, USA, was used throughout the study. For the preparation of mobile phase were used Methanol (MeOH, VWR BHD Prolabo, Singapore) and MQ water produced by dispenser Milli-Q Integral 3 Water Purification System, Merck Milliepore, Billerica, MA, USA.

### 2.2. Film Production

Films were produced by a casting/solvent evaporation technique using HPMC5 (8% *w*/*v*), and the plasticizer glycerol (2% *w*/*v*) with or without HP-β-CD (5% *w*/*v*).

HPMC5, Gly, and HP-β-CD were dissolved in 100 mL of 0.5 mg/mL furosemide solution in simulated saliva (phosphate buffer, pH 6.7, containing NaCl). The resulting solution was maintained under magnetic stirring for 5 h at room temperature and protected from light. The mixture was casted onto a Coatmaster 510 ERICHSEN GmbH and CO. KG, Hemer, Germany, equipped with the Wasag Model 288 Film Applicator System, with a gap opening of 1000 μm resulting in a wet film thickness of 1000 μm. The film was dried at room temperature for 15 h with the light switched off. The thin film was detached from the coatmaster and stored in an aluminium sachet to protect it from light.

The obtained OTFs were characterized for thicknesses using Cocraft 2.5 micrometer (0–25 mm). Uniformity of furosemide content was evaluated as follows. A large sample of OTF was cut into smaller pieces (dimensions 3 cm × 1 cm) and, each of them, was solubilized in 10 mL of simulated saliva (at room temperature) until complete dissolution. The solutions obtained were first diluted with EtOH (1:20 *v*/*v*) to obtain the dissociation of the inclusion complex and then analyzed in UV/Vis Spectrophotometer (UV-2401 PC Shimadzu Corporation, Kyoto, Japan) at λ = 276 nm.

### 2.3. Rheological Studies

Rheological experiments were carried out with a TA HR 2 stress-control Rotational Rheometer operated by software TRIOS (TA instruments, Waters Spa, Milan, Italy). Flow curves of all the film-forming solutions were obtained with a cone-plate geometry (diameter 40 mm, angle 1${}^{\circ }$) in the range of 0.01–1000 Pa at 25.0 ± 0.1 ${}^{\circ }$C. All the experiments were carried out at least in triplicate.

### 2.4. Mechanical Strength Tests

Mechanical strength tests were carried out on square OTFs pieces (2 cm × 2 cm) with a Texture Analyzer TA-XT plus (Stable Microsystems, Godalming, UK) using a cylindrical probe with a plane flat-faced surface (radius 3.52 mm).

The sample was fixed in the sample holder, and the probe was moved down at a speed of 1.0 mm/s. The measurement started when the probe came in contact with the film sample (trigger force 0.05 g). The probe moved at a constant test speed of 0.1 mm/s until the film ruptured, and the applied force and displacement (penetration depth) was registered. The puncture strength and elongation to break were calculated [16].

Puncture strength was calculated as

Puncture strength (N/mm${}^{2}$) = Puncture force (N)/Area of the probe (mm${}^{2}$) where the Puncture force is the maximum force recorded in the test.

Elongation to break was calculated according to the following equation

(1) $$Elongation\;to\;break=\left(\frac{\sqrt{a^{\prime 2}+b^{2}}+r}{a}-1\right)\times 100$$

where *a* is the radius of the film in the sample holder opening, i.e., initial length, $a^{\prime }$ is the initial length minus radius of probe, *b* is the penetration depth/vertical displacement by the probe, and *r* is the radius of the probe.

All measurements were conducted at room conditions and repeated for different pieces of each formulation (*n* = 20).

### 2.5. Phase Solubility Study of Furosemide with HP-β-CD

Phase solubility studies were carried out according to the method reported by Higuchi and Connors [17]. Excess amounts of furosemide were added to saliva simulata solutions (pH = 6.7) containing HP-β-CD in different concentrations (0%, 2%, 4%, 6%, 8%, 10% *w*/*v*) and shaken for 3 days at constant temperature (T = 37 ${}^{\circ }$C) in a cabinet shaker. The filtered solutions were diluted with EtOH (1:20 *v*/*v*) and analyzed on UV spectrophotometer at λ = 276 nm to define solubility characteristics.

### 2.6. Swelling-Erosion Tests

A square piece (1 cm × 1 cm) of dry film was weighted (initial weight $W_{0}$) and inserted in a dry beaker (tare weight $W_{b}$). Then, 1.5 mL of simulated saliva (pH 6.7) at 37 ${}^{\circ }$C ± 0.1 ${}^{\circ }$C was added on the film with a pipette to allow the film to swell and erode, taking great care not to wet the beaker. At regular time instants $t_{f}$, the excess of simulated saliva (not absorbed by the film) was carefully removed with a syringe, the system made by wet film + beaker was weighed (thus recording the total weight $W\left(t_{f}\right)+W_{b}$), and new fresh saliva was added to continue the test. From the difference between the total weight of the system and the weight of the beaker $W_{b}$, we estimated the weight of the swelling film at time $t_{f}$. The weight of the film increases until dissolution occurs and the film weight starts to decrease because the eroded material is removed together with the excess of simulated saliva.

### 2.7. Release Studies with Vertical Franz Diffusion Cell

Release studies were carried out at 37 ${}^{\circ }C$ using a jacketed Franz diffusion cell (PermeGear, Hellertown, PA, USA).

The cylindrical donor compartment had a cross-section area of 1 cm${}^{2}$. The donor chamber was separated from the receptor chamber by a dialysis membrane ($Spectra/Por^{®}$ 2, Standard RC Discs, MWCO 12–14 kDa, Spectrum® Laboratories, Rancho Dominguez, CA, USA) having an area of 1 cm${}^{2}$ and thickness $\delta_{m}$ = 45 μm.

The receptor chamber was filled with 7.9 mL of sonicated simulated saliva pH 6.7 at 37 ${}^{\circ }C$ under constant stirring (500 rpm).

Aliquots of 200 μL were withdrawn at fixed time intervals and replaced with equal volumes of fresh saliva.

For blank experiments, the donor compartment was loaded with (1) 0.1 mL of furosemide solution (0.18 mg/mL in simulated saliva) and (2) 0.1 mL of furosemide + HP-β-CD solution (0.18 mg/mL of furosemide + 5% *w*/*v* of HP-β-CD in simulated saliva). Blank experiments were performed for estimating the diffusion coefficient of furosemide $D_{0}^{f}$ and furosemide/HP-β-CD complex $D_{0}^{f+CD}$ in the solvent solution and in the dialysis membrane.

Release studies in the Franz cell were also performed by placing 1 cm${}^{2}$ of thin dry film (with or without HP-β-CD) in the donor compartment. The film, in contact with the solvent solution (simulated saliva in the receptor chamber) through the dialysis membrane, swells and erodes, but the eroded material is not washed away and removed but it remains in the donor compartment. The drug (furosemide or furosemide/HP-β-CD complex) is therefore released from the donor chamber to the receptor compartment. Release experiments from films in the Franz cell were performed to estimate the effective diffusion coefficient of furosemide $D_{G}^{f}$ and furosemide/HP-β-CD complex $D_{G}^{f+CD}$ in the swollen gel.

Furosemide was quantified by HPLC analysis carried out with a Shimadzu apparatus (Kyoto, Japan) equipped with Pump LC-20AD, Auto injector SIL-9A, Detector SPD-10A, Plotter Chromatopak C-R5A, Column oven IGLOO-CIL HPLC column thermostat, SPC GmbH, Separation column, C18, 4 μm, 3.9 × 150 mm Cartridge $Nova-Pak^{®}$ Waters, USA with a $Nova-Pak^{®}$ Guard Column C18, 4 μm, 3.9 × 20 mm.

The HPLC analysis was carried out using a mixture of MeOH and phosphate buffer (pH 6.8) (30:70, *v*:*v*) as the mobile phase, with a flow rate 1 mL/min and monitoring furosemide at λ = 276 nm.

### 2.8. Release Studies with Paddle Type Dissolution Apparatus (USP II)

A rotating paddle apparatus (USP II Prolabo Dissolution Tester, Sion, France) was used to test the release at 37 ${}^{\circ }$C and 50 rpm. OTFs were anchored with a wire mesh to the bottom of the vessel filled with 500 mL of preheated simulated saliva (pH 6.7). In this way, both film surfaces were exposed to the solvent. Aliquots (2 mL) of the release medium were withdrawn at fixed time intervals and replaced with equal volumes of fresh saliva. The solutions obtained were analyzed in UV/Vis Spectrophotometer (UV-2401 PC Shimadzu Corporation, Kyoto, Japan) at λ=276 nm. Tests were repeated in triplicate.

### 2.9. Release Studies with Millifluidic Flow-through Device (MFTD)

Drug release studies from thin films were also performed using the recently proposed millifluidic continuous flow-through device (henceforth referred to with the acronym MFTD) [14,15].

**Figure 1:**
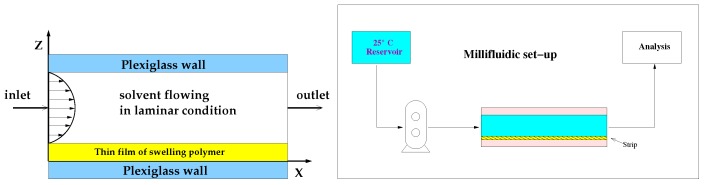
Millifluidic flow-through device. Schematic representation of the experimental set-up.

A schematic representation of the device and of the experimental set up are shown in Figure 1.

The device is called “millifluidic” because it has a characteristic operative volume in the order of mm${}^{3}$. The millifluidic device has been designed to mimic mouth physiological conditions because of the laminar tangential solvent flow, the flow rates comparable to salivary flow rates and low hold-up volume.

In the MFTD, thin film strips were placed on the bottom plate of a dissolution cell with dimensions of 2 × 9 × 30 mm. These dimensions were chosen to assure a regular flow through the device also after complete film swelling. The surface area of the OTF exposed to the solvent tangential laminar flow was 9 × 30 mm.

One side of the film was exposed to the tangential solvent flow, the other side in contact with the bottom wall of the dissolution cell. As soon as wetted, strips adhered firmly to the plate, and there was no need to make use of a double-sided tape, thus avoiding unpredictable and ruinous detachments or floating problems often encountered with other existing devices (USP I, USP II).

Dissolution medium (simulated saliva) was kept in a reservoir at 37 ± 1 ${}^{\circ }$C and circulated through the dissolution cell in open loop, by means of a volumetric pump (see Figure 1). Flow rates investigated in this work were in the range Q∈ [1–5] mL/min corresponding to laminar flow conditions with Reynolds numbers $Re=\rho <v>d_{e}/\mu \in$ [1–20], $d_{e}$ being the hydraulic radius $d_{e}=$ 4 × cross-section area/wetted perimeter = 3.27 mm. Flow rates investigated were comparable with salivary flow rates Q∈ [1–4] mL/min.

Solution coming out the cell was sent to the UV/Vis analyzer (UV-2401 PC, Shimadzu Corporation, Kyoto, Japan, continuous flow cell, optical path 1 mm) in order to quantify the amount of active ingredient released from the swelling film. Drug concentration values $c_{s}\left(t\right)$ mg/mL were recorded every 2 s. The amount of drug released was calculated with a calibration curve. Calibration curve for furosemide reference standard (RS) was obtained by measuring the UV absorption (λ = 276 nm) in dissolution medium (simulated saliva). The linearity of the calibration curves was confirmed in the range 1–100 μg/mL with a regression coefficient (R${}^{2}$) value of 0.999. Limits of detection and quantification are 0.2 μg/mL. Tests were repeated in triplicate.

The differential F(t) and integral $M_{t}$ release curves were subsequently computed from the experimental data as follows

(2) $$F\left(t\right)=Q\;c_{s}\left(t\right)\;,\;M_{t}=\int_{0}^{t}Qc_{s}\left(t^{\prime }\right)dt^{\prime }=\int_{0}^{t}F\left(t^{\prime }\right)dt^{\prime }$$

$t_{f}$ being a final time for the experimental test sufficiently long to ensure the complete drug release. The final time $t_{f}$ was changed according to the flow rate *Q*. The smaller the flow rate *Q*, the longer the time interval required for complete drug release.

## 3. Transport Models

In this section, we present all the mathematical models we adopted for the analysis of experimental data of swelling-erosion tests, and the drug release in the Franz cell, in the millifluidic flow-through device and in the USP II apparatus.

### 3.1. Swelling-Erosion Modeling of Thin Films

We adopted a 1D model of swelling and erosion of thin films. Swelling along the *z* direction (orthogonal to the x−y plane representing the flat surface of the OTF) is a moving boundary problem. The solvent mass balance equation, written in terms of the solvent, volumetric fraction ϕ reads as [13,15,18,19]

(3) $$\frac{\partial \phi }{\partial t}=\frac{\partial }{\partial z}\left(D^{s}\left(\phi \right)\left(1-\phi \right)\frac{\partial \phi }{\partial z}\right),\;R\left(t\right)<z<S\left(t\right),\;t>0$$

where S(t) and R(t) are, respectively, the position of the erosion front (gel–solvent interface) and swelling front (glassy–rubbery interface) at time *t* coming out the cell. $D^{s}\left(\phi \right)$ is the solvent diffusion coefficient, modeled as an increasing function of the solvent volumetric fraction ϕ

(4) $$D^{s}\left(\phi \right)=D_{G}^{s}\exp\left[-\beta_{s}\left(\frac{\phi -\phi_{eq}}{\phi_{0}-\phi_{eq}}\right)\right]\;,\;\beta_{s}\geq 0$$

where $D_{G}^{s}$ is the solvent diffusion coefficient in the fully swollen gel, $\phi_{eq}$ and $\phi_{0}$ are the solvent volumetric fraction at equilibrium and in the dry film, respectively. If $\beta_{s}=0$, then the solvent diffusion coefficient $D^{s}$ is assumed constant and equal to $D_{G}^{s}$.

On the glassy-rubbery front R(t), a threshold concentration to initiate swelling $\phi_{g}>\phi_{0}$ is assumed [20] and the front movement is given by the Stefan condition

(5) $$\phi =\phi_{g},\;\left(\phi_{g}-\phi_{0}\right)\frac{dR}{dt}=-D^{s}\left(\phi_{g}\right)\left(1-\phi_{g}\right){\left.\frac{\partial \phi }{\partial z}\right|}_{R(t)}\;\;at\;z=R\left(t\right).$$

When R(t) reaches z=0, the glassy phase disappears and the no-flux boundary condition applies

(6) $$\frac{\partial \phi }{\partial z}=0\;at\;z=0.$$

On the gel-solvent interface S(t), thermodynamic equilibrium $\phi_{eq}$ is assumed and the front movement is described by the Stefan condition

(7) $$\phi =\phi_{eq},\;\frac{dS}{dt}=D_{G}^{s}{\left.\frac{\partial \phi }{\partial z}\right|}_{S(t)}-R_{dis}\;\;at\;z=S\left(t\right)$$

where $R_{dis}$ is the disentanglement rate accounting for erosion [18,19,21,22,23,24]

(8) $$R_{dis}=C\;{\left(1-\phi \right)}^{1.625}$$

where *C* [μm/s] is a constant depending on the fluid-dynamic conditions.

The no-flux boundary condition at z=0, Equation (6), represents a symmetry boundary condition when both film surfaces are exposed to the solvent like in a swelling test or in a release experiment in USP apparatuses. Correspondingly, the moving boundary problem has to be solved with initial conditions

(9) $$R\left(0\right)=S\left(0\right)=L_{0}/2\;,\;\phi \left(z,0\right)=\phi_{0}\;,\;0\leq z\leq L_{0}/2$$

where $L_{0}/2$ is the half thickness of the dry film.

If film swelling occurs in the millifluidic device, Equation (6) represents an impermeability condition since the thin film adheres firmly on the bottom wall of the device and no solvent permeation is allowed at the bottom surface of the film. Therefore, the moving boundary model describing film swelling and erosion in the MFTD must be solved with initial conditions

(10) $$R\left(0\right)=S\left(0\right)=L_{0}\;,\;\phi \left(z,0\right)=\phi_{0}\;,\;0\leq z\leq L_{0}$$

where $L_{0}$ is the initial thickness of the dry film.

The swelling-erosion model presented is a nonlinear moving boundary model that must be necessarily numerically solved and therefore no explicit equations for the time evolution of the glassy-rubbery interface R(t) or for the rubbery-solvent interface S(t) are available. When modeling drug release in different devices (USP II apparatus and MFTD), the drug transport equations must be solved together with the moving-boundary model equations describing the swelling-erosion dynamics, that furnish, at each time instant, front positions R(t) and S(t).

### 3.2. Drug Release Modeling in a Vertical Franz Diffusion Cell

Drug release data obtained with a vertical Franz diffusion cell can be analyzed by adopting different transport models with increasing complexity.

**Model I.** The simplest model assumes that the drug is uniformly distributed in both the donor chamber (volume $V_{d}$, concentration $c_{d}\left(t\right)$) and in the accepting compartment (volume $V_{res}$ perfectly mixed, concentration $c_{res}\left(t\right)$). Donor and accepting chambers are separated by a membrane with area *A* and thickness $\delta_{m}$. Therefore, the macroscopic balance equation of drug in the donor chamber reads as

(11) $$V_{d}\frac{dc_{d}}{dt}=-\frac{D_{m}^{d}}{\delta_{m}}\;A\;\left(c_{d}-c_{res}\right)\;,\;c_{d}\left(0\right)=c_{0}\;$$

where $c_{0}$ is the initial drug concentration in the donor compartment, and $D_{m}^{d}$ is the drug diffusivity in the membrane, which can be assumed equal or lower than drug diffusivity $D_{0}^{d}$ in the solvent solution loaded in the receptor chamber. This intrinsically depends on the drug molecular weight and the membrane cutoff.

By assuming perfect sink conditions $c_{res}\left(t\right)=0$, the amount of drug released up to time *t* attains the form

(12) $$M_{t}=V_{d}c_{0}-V_{d}c_{d}\left(t\right)=M_{0}\left(1-\exp\left(-\frac{D_{m}^{d}A}{\delta_{m}V_{d}}\;t\right)\right).$$

This model neglects drug concentration gradients in the donor compartment and is extremely unreliable.

**Model II.** A more accurate model accounts for drug concentration gradients along the vertical direction *z* in both the donor chamber $-\delta_{d}\leq z\leq 0$ and in the membrane $0\leq z\leq \delta_{m}$. The membrane is placed at z=0, the *z* axis is oriented towards the bottom, and $\delta_{d}$ is the thickness of the solvent solution placed in the donor compartment.

By adopting a purely diffusive transport equation for drug concentration $c_{d}\left(z,t\right)$, the model equations read as

(13) $$\begin{array}{ccc} \frac{\partial c_{d}}{\partial t} & = & D^{d}\frac{\partial^{2}c_{d}}{\partial z^{2}}\;,\;c_{d}\left(z,0\right)=c_{0}\;,\;-\delta_{d}<z<0 \end{array}$$

(14) $$\begin{array}{ccc} \frac{\partial c_{d}}{\partial t} & = & D_{m}^{d}\frac{\partial^{2}c_{d}}{\partial z^{2}}\;,\;c_{d}\left(z,0\right)=0\;,\;0<z<\delta_{m} \end{array}$$

(15) $$\begin{array}{ccc} {\left.\frac{\partial c_{d}}{\partial z}\right|}_{x=-\delta_{h}} & = & 0\;,\;{\left.D^{d}\;\frac{\partial c_{d}}{\partial z}\right|}_{z=0^{-}}=D_{m}^{d}{\left.\frac{\partial c_{d}}{\partial z}\right|}_{z=0^{+}}\;,\;c_{d}\left(\delta_{m},t\right)=c_{res}\left(t\right) \end{array}$$

where $D^{d}$ is the drug diffusivity in the solution placed in the donor compartment.

At this point, we can distinguish between three different levels of model accuracy.

**Model IIa.** By assuming perfect sink conditions, model Equations (13)–(15) can be solved with the further condition $c_{res}\left(t\right)=0$, and $M_{t}$ can be evaluated, at each time instant *t*, as

(16) $$M_{t}=M_{0}-A\int_{-\delta_{h}}^{\delta_{m}}c_{d}\left(z^{\prime },t\right)dz^{\prime }.$$

**Model IIb.** By accounting for the finite volume $V_{res}$ of the accepting chamber, the concentration $c_{res}\left(t\right)$, still assumed uniform in the accepting chamber, evolves in time according to the macroscopic balance equation

(17) $$V_{res}\frac{dc_{res}}{dt}=-D_{m}^{d}\;A\;{\left.\frac{\partial c_{d}}{\partial z}\right|}_{z=\delta_{m}}\;,\;c_{res}\left(0\right)=0$$

that must be solved together with the transport Equations (13)–(15). Correspondingly, the amount of drug released up to time *t* can be computed from Equation (16) or equivalently as

(18) $$M_{t}=V_{res}\;c_{res}\left(t\right).$$

**Model IIc.** It should be observed that, during the experiment, one performs withdrawals (volume $V_{p}$ at specific time instants $t_{i}$) from the accepting chamber, and this influences the diffusion process, since each withdrawal reduces almost instantaneously the concentration $c_{res}$ of a quantity $\Delta c_{res}=c_{res}\left(1-V_{p}/V_{res}\right)$. The withdrawal does not alter $V_{res}$ if an equal volume $V_{p}$ of solvent is replaced just after the withdrawal.

The assumption of perfect mixing in the accepting chamber implies $c_{p}\left(t_{i}\right)=c_{res}\left(t_{i}\right)$ and the effect of withdrawals can be accounted for in the balance equation for $c_{res}$ as follows:

(19) $$V_{res}\frac{dc_{res}\left(t\right)}{dt}=-D_{m}\;A\;{\left.\frac{\partial c_{d}}{\partial z}\right|}_{z=\delta_{m}}-\sum_{j=1}^{N_{i}\left(t\right)}V_{p}\;c_{p}\left(t_{j}\right)$$

where $N_{i}\left(t\right)$ is the number of withdrawals performed from t=0 up to time *t*.

By solving the transport Equations (13)–(15) together with Equation (19), $M_{t}$ can be evaluated as

(20) $$M_{t}=V_{res}\;c_{res}+\sum_{j=1}^{N_{i}}V_{p}c_{p}\left(t_{j}\right).$$

By focusing on Equation (20), one can observe that it represents the usual way the experimental integral release curve is evaluated from the experimental differential release curve (represented by withdrawal concentrations $c_{p}\left(t_{i}\right)$ at withdrawal time instants $t_{i}$)

(21) $$M_{t}^{s}\left(t_{i}\right)=V_{res}c_{p}\left(t_{i}\right)+\sum_{j=1}^{N_{i}\left(t_{i}\right)}V_{p}c_{p}\left(t_{j}\right)$$

if one assumes perfect mixing in the donor chamber so that $c_{res}\left(t_{i}\right)=c_{p}\left(t_{i}\right)$.

Therefore, experimental integral release curves—as usually reported in the whole literature—are intrinsically based on the assumption that the acceptor chamber is perfectly mixed and that the withdrawal concentration $c_{p}\left(t_{i}\right)$ is representative of the (uniform) concentration $c_{res}\left(t\right)$ at the withdrawal time instant $t_{i}$.

**Model III.** In order to verify the validity of the hypothesis of perfect mixing, we performed a release experiment in an unjacketed Franz cell with a colored marker and investigated, by visual inspection, the actual mixing in the receptor chamber during the release process.

We choose to adopt an unjacketed Franz cell (with dimensions very close to the jacketed Franz cell adopted for furosemide release experiments) in order to have a better visualization of the mixing/diffusion process occurring within the receptor chamber and to identify which portion of it is actually “well mixed”. Experiments are performed at laboratory temperature $T≃25{\;}^{\circ }$C with KMnO${}_{4}$ 0.1 M in distilled water.

**Figure 2:**
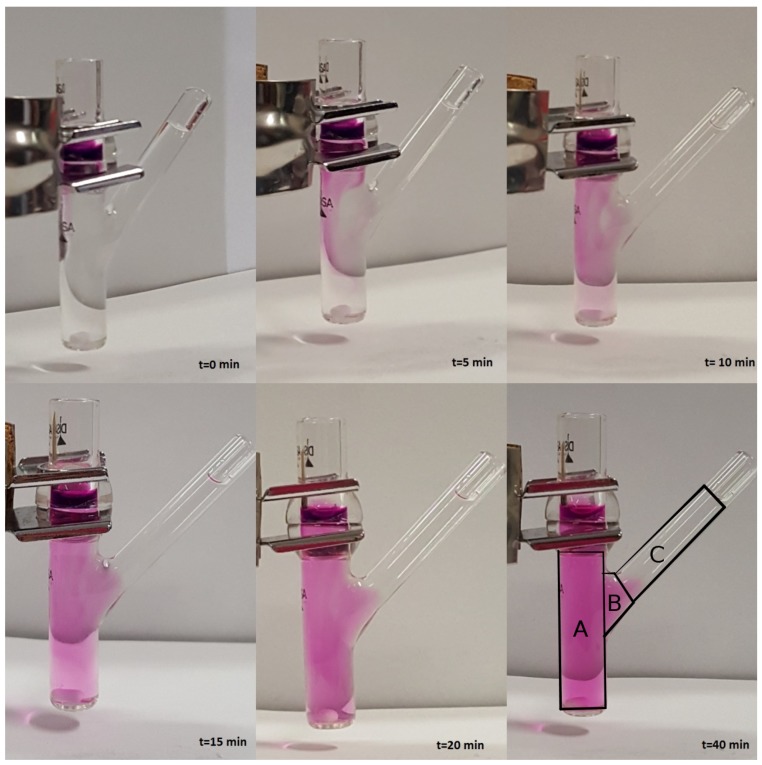
Release experiment in an unjacketed Franz cell at $T=25{\;}^{\circ }$C with a colored marker (KMnO${}_{4}$ 0.1 M in distilled water). Rotational speed: 100 rpm.

**Figure 3:**
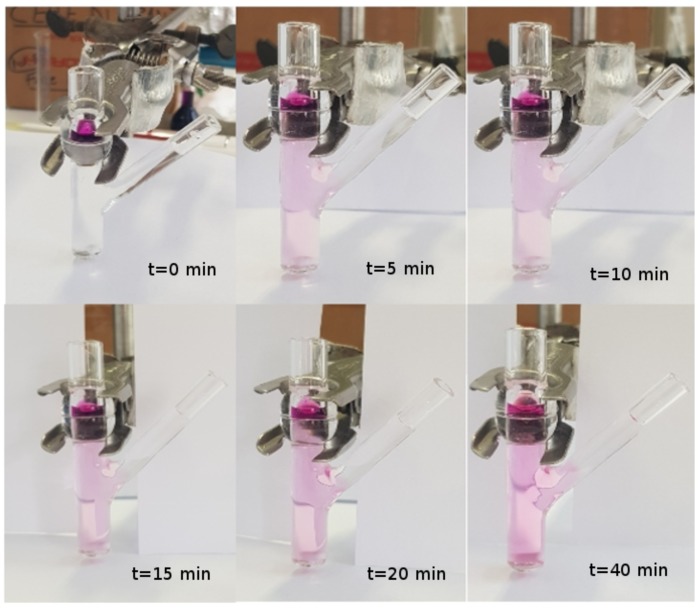
Release experiment in an unjacketed Franz cell at $T=25{\;}^{\circ }$C with a colored marker (KMnO${}_{4}$ 0.1 M in distilled water). Rotational speed: 500 rpm. The reason why the color is much fader in this experiment as compared to that with 100 rpm—the concentration of KMnO${}_{4}$ being the same—is only a matter of colors in the photo (different camera and different light exposition).

We investigated two different rotational speeds, namely 100 (Figure 2) and 500 rpm (Figure 3). Rotational speeds of 100–200 rpm are usually adopted because they do not create problems due to the formation of micro bubbles that can float, rise, and stratify close to the dialysis membrane. The rotational speed of 500 rpm is extremely high. It guarantees a very good mixing (at least in the cylindrical part of the receiving compartment) but requires a preliminary sonication of the solvent solution (simulated saliva) in order to minimize bubble formation.

From Figure 2 and Figure 3, it can be clearly observed that the lateral arm is almost unaffected by mixing induced by the magnetic stirrer. In fact, the dye concentration in the lateral arm is negligible or at least significantly lower than that in the main stirred body of the receptor chamber for both rotational speeds. Moreover, it can be clearly identified a “color/concentration transition zone” at the connection between the main cylindrical body (well mixed, especially at 500 rpm) and the later arm (totally unmixed).

**Figure 4:**
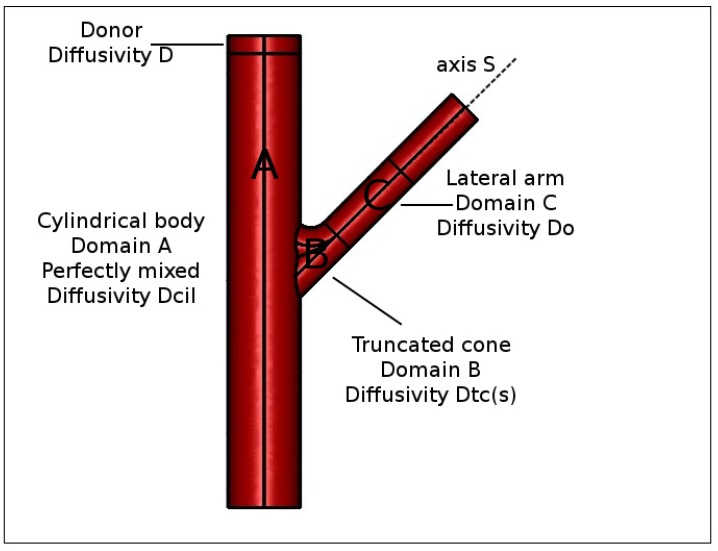
Schematic representation of different domains in a Franz cell. Donor compartment, drug diffusivity $D^{d}$. (A) Cylindrical body, perfectly mixed, drug diffusivity $D_{cil}^{d}=\left(10^{5}÷10^{7}\right)\times D_{0}^{d}$. (B) Truncated cone region, drug diffusivity $D_{tc}^{d}\left(s\right)$, Equation (22). (C) Lateral arm, drug diffusivity $D_{0}^{d}$.

For this reason, we were forced to abandon the simplifying hypothesis of perfect mixing of the accepting chamber and to distinguish within it three different domains (see Figure 4) characterized by different fluid dynamic regimes.

**Domain 1.** A cylindrical body (Domain A, volume $V_{cil}≃7.1$ mL), in the bottom of which is placed the magnetic stirrer, that we reasonably can assume perfectly mixed (especially for high rotation speeds) or equivalently characterized by an extremely high drug diffusion coefficient $D_{cil}^{d}=\left(10^{5}÷10^{7}\right)D_{0}^{d}$, $D_{0}^{d}$ being the drug diffusivity in the unstirred solvent solution.

**Domain 2.** A lateral arm (Domain C, volume $V_{arm}≃$ 0.5–0.6 mL) that is not reached by vortices generated by the magnetic stirrer and in which we assume a purely diffusive drug transport mechanism with diffusivity $D_{0}^{d}$.

**Domain 3.** A third domain similar to a truncated cone (Domain B, volume $V_{tc}≃$ 0.2–0.3 mL), connecting the cylindrical body to the lateral arm, where the syringe carries out withdrawals and solvent reintegrations. In this connection domain, we can assume that the drug diffusion coefficient is position-dependent.

If we introduce a curvilinear abscissa *s* (see Figure 4), we can assume that the drug diffusion coefficient exponentially decreases from $D^{d}=D_{cil}^{d}$ for s=0, i.e., in contact with the well mixed cylindrical body, to $D^{d}=D_{0}^{d}$ when the drug enters the lateral arm

(22) $$D_{tc}^{d}\left(s\right)=D_{0}^{d}+\left(D_{cil}^{d}-D_{0}^{d}\right)\exp\left(-\beta_{D}s\right)\;with\;\beta_{D}≃500\;m^{-1}.$$

Equation (22) permits us to model drug transport in Domains B and C (total volume $V_{tc}+V_{arm}≃0.8$ mL) as a pure diffusive process with a space-dependent diffusion coefficient.

The model we implemented thus describes drug release in a Franz cell with

1. a purely diffusive transport equation in the donor compartment with drug diffusivity $D^{d}$,
2. a purely diffusive transport equation in the membrane with drug diffusivity $D_{m}^{d}\leq D_{0}^{d}$,
3. a purely diffusive transport equation in the cylindrical body (Domain A) with drug diffusivity $D_{cil}^{d}>>D_{0}^{d}$,
4. a purely diffusive transport equation in the truncated cone region (Domain B) with diffusivity $D_{tc}^{d}\left(s\right)$, Equation (22), and
5. a pure diffusive transport equation in the lateral arm (Domain C) with drug diffusivity $D_{0}^{d}$ in a quiescent solvent solution.

Continuity of drug concentration and diffusive fluxes are enforced at all internal boundaries (boundaries between two distinct domains) and zero flux boundary condition at the top of the donor compartment and at the exit of the lateral arm are assumed from the assumption of negligible solvent evaporation.

The split of the accepting chamber into three distinct domains led us to a more accurate evaluation of the withdrawal concentration $c_{p}\left(t_{i}\right)$ as the average drug concentration $\bar{c_{tc}}$ in the truncated cone (Domain B) at time $t_{i}$

(23) $$c_{p}\left(t_{i}\right)=\bar{c_{tc}}=\frac{1}{V_{tc}}\int_{V_{tc}}c_{d}\left(x,t_{i}\right)dx\;.$$

For the sake of simplicity, we assume that the volume of truncated cone domain $V_{tc}$ equals the withdrawal volume $V_{p}$. In this way, the influence of the withdrawal and subsequent reintegration of solvent solution can be simply modeled as an instantaneous complete depletion of drug concentration in the truncated cone (Domain B) at the withdrawal time instants $t_{i}$.

A correct estimate of the drug diffusion coefficient in the donor compartment *D* can therefore be obtained by a direct comparison between experimental data of withdrawal concentrations $c_{p}\left(t_{i}\right)$ and model predictions (Equation (23)).

In point of fact, the integral release curve accounting for non-perfect mixing

(24) $$\begin{array}{ccc} M_{t} & = & \int_{V_{cil}}c_{d}\left(x,t_{i}\right)dx+\int_{V_{tc}}c_{d}\left(x,t_{i}\right)dx+\int_{V_{arm}}c_{d}\left(x,t_{i}\right)dx+\sum_{j=1}^{N_{i}}V_{p}c_{p}\left(t_{j}\right)\\ & = & V_{cil}\bar{c_{cil}}+V_{tc}\bar{c_{tc}}+V_{arm}\bar{c_{arm}}+\sum_{j=1}^{N_{i}}V_{p}\bar{c_{tc}}\left(t_{j}\right) \end{array}$$

will necessarily differ from the “experimental” integral release curve Equation (21) based on the assumption of perfect mixing, i.e., $\bar{c_{cil}}=\bar{c_{tc}}=\bar{c_{arm}}=c_{res}$. In particular, the integral release curve $M_{t}^{s}$ based on perfect mixing assumption tends to underestimate $M_{t}$ since $\bar{c_{tc}}<\bar{c_{cil}}$.

### 3.3. Drug Release Modeling in the MFTD

The drug release process from the OTFs in the millifluidic device is described with a 2D model. Let *x* be the axial flow direction, spanning the channel length $0\leq x\leq L_{x}$, $L_{x}=30$ mm. Let *z* be the vertical direction (preferential swelling direction) $0\leq z\leq L_{z}$, $L_{z}$ being the channel thickness $L_{z}=2$ mm. Let $L_{y}$ be the channel width $L_{y}=0.9$ mm. The thin film exposed to the solvent tangential flow is a thin strip of surface $L_{x}\times L_{y}$ and initial thickness $L_{0}<<L_{z}$.

Let $c^{G}\left(x,z,t\right)$ and $c^{F}\left(x,z,t\right)$ be the drug concentration in the swelling film and in the flow channel, respectively.

Swelling-erosion dynamics is described by the 1D model presented in Section 3.1 and evolves independently of the drug release process. The swelling model furnishes, at each time instant *t*, the gel–solvent S(t), and the glassy-rubbery R(t) front positions.

Drug balance equation in the gel layer reads as

(25) $$\frac{\partial c^{G}}{\partial t}=D_{G}^{d}\;\nabla^{2}c^{G},\;0<x<L_{x},\;R\left(t\right)<z<S\left(t\right)$$

where $D_{G}^{d}$ is the effective drug diffusivity in the swollen gel.

Equation (25) must be solved with the boundary condition of impermeable wall at $x=0,L_{x}$ and Stefan condition at glassy-rubbery interface z=R(t):

(26) $$\frac{dR(t)}{dt}\left(c_{0}-c^{G}\right){|}_{x,R(t)}=D_{G}^{d}\frac{\partial c^{G}}{\partial z}{|}_{x,R(t)}$$

where $c_{0}$ is the initial drug loading in the dry film. When R(t) reaches z=0, then dR/dt=0 and consequently Equation (26) transforms into the impermeability condition at the bottom wall z=0 of the device. Initial conditions are

(27) $$c^{G}\left(x,z,0\right)=c_{0}\;for\;0\leq z\leq L_{0},\;\forall x,\;S\left(0\right)=R\left(0\right)=L_{0}.$$

The drug balance equation in the solvent (flowing in laminar flow conditions) is a convection–diffusion transport equation

(28) $$\frac{\partial c^{F}}{\partial t}=D_{0}^{d}\nabla^{2}c^{F}-v_{x}\left(z,t\right)\frac{\partial c^{F}}{\partial x},\;0<x<L_{x},\;S\left(t\right)<z<L_{z}$$

where $D_{0}^{d}$ is the drug diffusivity in the solvent solution, and $v_{x}\left(z,t\right)$ is the velocity of the solvent in the channel:

(29) $$v_{x}\left(z,t\right)=\frac{6Q\left(z-S\left(t\right)\right)\left(L_{z}-z\right)}{L_{y}{\left(L_{z}-S\left(t\right)\right)}^{3}},\;\int_{S(t)}^{L_{z}}v_{x}\left(z^{\prime },t\right)dz^{\prime }=\frac{Q}{L_{y}}$$

where the parabolic axial velocity field $v_{x}\left(z,t\right)$ is evaluated from lubrication theory by enforcing a constant flow rate and no slip boundary conditions and evolves in time according with the gel–solvent interface dynamics.

Equation (28) has to be solved with initial conditions

(30) $$c^{F}\left(x,z,0\right)=0\;for\;S\left(0\right)\leq z\leq L_{x},\;\forall x,\;S\left(0\right)=R\left(0\right)=L_{0}$$

and boundary conditions representing Danckwertz conditions at the channel outlet and no flux condition at the top impermeable channel wall

(31) $$c^{F}{|}_{0,z}=0\;,\;\frac{\partial c^{F}}{\partial x}{|}_{L_{x},z}=0\;,\;\frac{\partial c^{F}}{\partial z}{|}_{x,L_{z}}=0.$$

The drug concentration profiles in the channel and in the swollen gel are connected by the continuity condition at the gel-solvent interface S(t):

(32) $$c^{F}{|}_{x,S(t)}=c^{G}{|}_{x,S(t)},\;D_{0}^{d}\frac{\partial c^{F}}{\partial z}{|}_{x,S(t)}=D_{G}^{d}\frac{\partial c^{G}}{\partial z}{|}_{x,S(t)}.$$

Model results and experimental data can be compared in terms of differential release curves F(t) vs. *t* and integral release curves $M_{t}$ vs. *t* by computing

(33) $$F\left(t\right)=L_{y}\int_{S(t)}^{L_{z}}v_{x}\left(z^{\prime },t\right)c^{F}\left(L_{x},z^{\prime },t\right)dz^{\prime }\;,\;M_{t}=\int_{0}^{t}F\left(t^{\prime }\right)dt^{\prime }.$$

### 3.4. Drug Release Modeling in the USP II Apparatus

The drug release process from the OTFs in the USP II apparatus can be simply modeled by a 1D diffusive transport equation describing drug transport in the swelling film along the preferential swelling direction *z* (orthogonal to the flat surface of the thin film)

(34) $$\frac{\partial c^{G}}{\partial t}=D_{G}^{d}\frac{\partial^{2}c^{G}}{\partial z^{2}},\;R\left(t\right)<z<S\left(t\right)$$

to be solved with the equations describing the swelling-erosion dynamics (presented in Section 3.1) that furnishes, at each time instant, the gel-solvent S(t) and the glassy-rubbery R(t) front positions.

Equation (34) must be solved with the Stefan condition at the glassy-rubbery interface z=R(t):

(35) $$\frac{dR(t)}{dt}\left(c_{0}-c^{G}\right){|}_{R(t)}=D_{G}^{d}\frac{\partial c^{G}}{\partial z}{|}_{R(t)}.$$

When R(t) reaches z=0, then dR/dt=0 and consequently Equation (35) transforms into the symmetry condition at z=0, since both surfaces of the thin film are exposed to the solvent solution and the initial condition is therefore $S\left(0\right)=R\left(0\right)=L_{0}/2$, $L_{0}/2$ being the half thickness of the dry film.

In this device, given the large volume of solvent solution (500 mL) and the good mixing induced by paddle rotation, we can reasonably assume a perfect sink condition, thus enforcing at each time instant $c^{G}=0$ at z=S(t).

The total amount of drug released up to time *t* can be evaluated as

(36) $$M_{t}=A\left(c_{0}L_{0}-2\int_{R(t)}^{S(t)}c^{G}\left(z^{\prime },t\right)dz^{\prime }\right)$$

A being the thin dry film surface area.

## 4. Results and Discussion

### 4.1. Film Thickness, Drug Loading, and Moisture Content

Thicknesses of OTFs were in the range 70–95 μm for OTFs without HP-β-CD and 110–140 μm for OTFs with HP-β-CD.

The furosemide content in dry films (with and without HP-β-CD) was 61±8 μg/cm${}^{2}$ of film.

The residual water weight fraction (with and without HP-β-CD) was about 12–14% *w*/*w*.

### 4.2. Analysis of Rheological Studies and Mechanical Strength Tests

HPMC OTFs were produced by the solvent casting technique which involves the initial deposition and successive spreading of the polymeric solution on a solid support. The quality and properties of the final product are critically dependent on the viscosity of the starting polymeric solution.

If the viscosity is too low, the formulation will flow off the glass plate of the Coatmaster, whereas, if the viscosity is too high, the spreading with the knife will be problematic and the air bubbles will not be removed and leaves structures or holes in the dry film. The viscosity of 8% *w*/*v* HPMC was preliminary selected based on trial and errors and was found to be ideal for the preparation of thin films with the Coatmaster instrument.

It was important to perform rheological studies in order to evaluate the effect of glycerol and, above all, of HP-β-CD on the viscosity of the film-forming solutions [25].

The flow curves (Figure S1) show the same pseudoplastic behavior and almost the same viscosity for HPMC alone and for HPMC with glycerol and HP-β-CD. Therefore, the polymeric solution resulted rheologically stable and its viscosity substantially unaffected by the inclusion of glycerol and HP-β-CD.

Similar results were observed for the corresponding dried films which show comparable resistance and elasticity also in the presence of high amount of HP-β-CD (Figure S2).

### 4.3. Analysis of Phase Solubility of Furosemide with HP-β-CD

**Figure 5:**
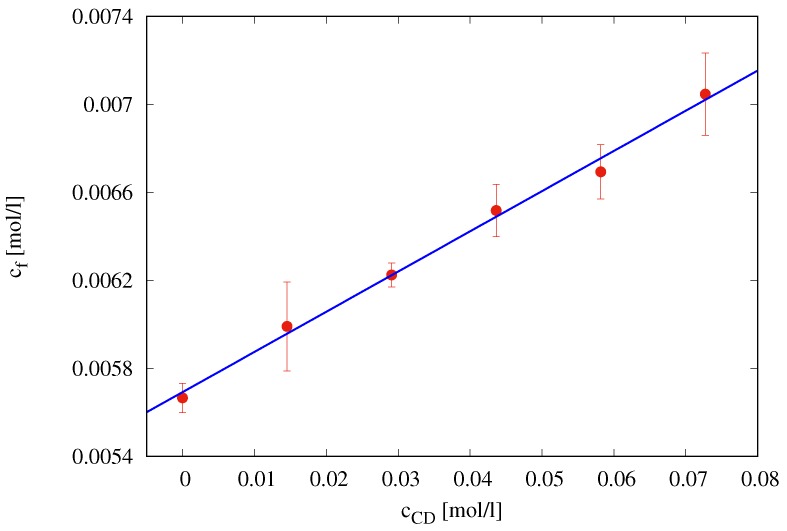
Phase solubility diagram of furosemide with HP-β-CD in simulated saliva (pH = 6.7) at $T=37{\;}^{\circ }$C.

The phase solubility plot, i.e., furosemide concentration at saturation $c_{f}$ [mol/L] vs. cyclodextrin concentration $c_{CD}$ [mol/L], is shown in Figure 5. It shows an AL type solubility curve [17].

The linear behavior

(37) $$c_{f}=\alpha +\beta \;c_{CD}\;,\;\alpha =0.0057\;\left(mol/L\right)\;,\;\beta =0.0183\;\left(ad\right)$$

characterized by a slope β significantly lower than unity indicates the formation of a 1:1 complex furosemide/HP-β-CD.

According to this hypothesis, a complexation equilibrium constant $K_{1:1}$

(38) $$K_{1:1}=\frac{\beta }{\alpha (1-\beta )}=3.27\;\left[{\left(mol/L\right)}^{-1}\right]$$

has been estimated as in Brewster and Loftsson [26]. Therefore, we can assume that, in the presence of an excess of HP-β-CD, all the furosemide appears to be complexed in a 1:1 furosemide/ HP-β-CD complex.

### 4.4. Analysis of Swelling-Erosion Tests

**Figure 6:**
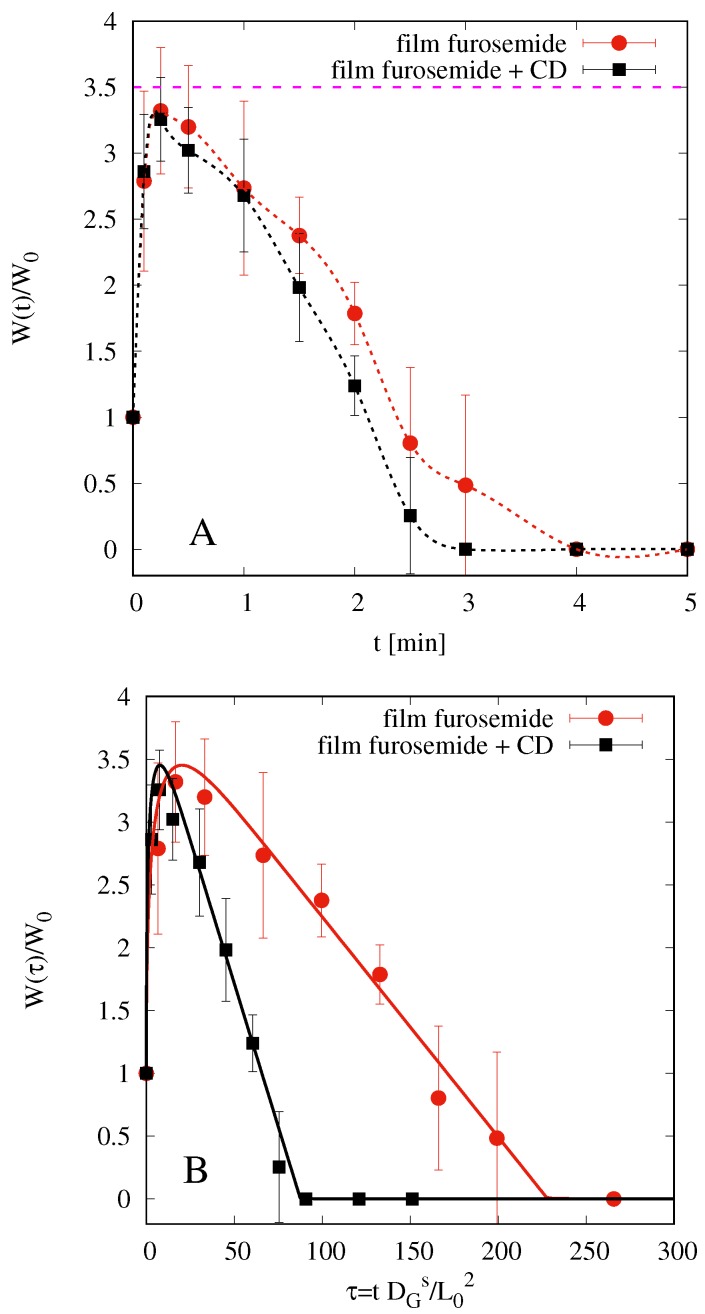
Swelling-erosion tests. (**A**) Experimental data $W\left(t\right)/W_{0}$ vs. *t* (min). (**B**) Experimental data and model predictions $W\left(\tau \right)/W_{0}$ vs. the dimensionless time τ. The maximum swelling degree is $W^{max}/W_{0}≃3.5$ with and without HP-β-CD.

Figure 6A shows the results of swelling tests for both films, with and without HP-β-CD. $W\left(t\right)/W_{0}$ represents film weight at time *t* rescaled onto the initial weight of the dry film.

We observe that both films start to swell, reach a maximum weight (approximately 3.5 times the initial one), and then begin to dissolve. Dissolution is very fast and almost complete in 4 min for film without HP-β-CD and in 3 min for film including HP-β-CD.

By adopting the swelling-erosion model presented in Section 3.1, we can estimate the solvent diffusion coefficient $D_{G}^{s}$ and the erosion constant *C*. Model parameters $\phi_{0}$, $\phi_{g}$, and $\phi_{eq}$ adopted for both films are reported in Table 1 together with best fit parameters $D_{G}^{s}$ and *C*.

In particular, $\phi_{0}$ is estimated from residual water weight fraction 12–14% *w*/*w* in the dry film as obtained from uniformity of content data. The glassy-rubbery threshold $\phi_{g}$ is assumed to be 10–15% greater than $\phi_{0}$, and $\phi_{eq}$ has been estimated from experimental data in order to obtain a maximum swelling $W^{max}/W_{0}≃3.5$.

The best fit value of the solvent diffusion coefficient $D_{G}^{s}=7.9\times 10^{-9}$ m${}^{2}$/s is in perfect agreement with the corresponding value obtained for simulated saliva in film made of pure HPMC K15M, as reported in [15]. In [15], no plasticizer was added, and the erosion effect was negligible. In the present case, the presence of glycerol as plasticizer may be responsible, together with a different viscosity grade between HPMC5 (used in the present paper) and HPMC K15M, of the significant erosion effect observed.

Figure 6B shows the comparison between experimental data and model predictions (continuous lines) in terms of the dimensionless time $\tau =tD_{G}^{s}/L_{0}^{2}$, i.e., the physical time rescaled onto the diffusion time that takes into account film thickness (significantly larger for films with HP-β-CD). This representation better highlights that the erosion effect is larger for films with HP-β-CD, as confirmed from best fit values for the erosion constant *C* for film with and without HP-β-CD.

It should be noticed that swelling-erosion data have been mainly used in order to estimate $\phi_{eq}$ and $D_{G}^{s}$ and not the disentanglement rate $R_{dis}$, because $R_{dis}$ from these experiments is largely overestimated. In fact, when the film swells and begins to dissolve, it splits into smaller pieces that can be removed from the beaker when the excess of simulated saliva is removed. This is evident if one compares the time scales for complete dissolution (3–4 min) in the swelling-erosion test and the time scale for complete drug release in USP II and in MFTD (10–20 min, depending on the fluid dynamic conditions). Therefore, a more accurate estimate of the disentanglement rate $R_{dis}$ can be obtained by a correct modelization of drug release experiments that are strongly influenced by erosion effects (see Section 4.6).

From the maximum swelling $W^{max}/W_{0}$, we can estimate the thickness of swollen films when inserted in the donor compartment of the Franz cell (see Section 4.5.2).

### 4.5. Analysis of Release Kinetics in Franz Cell

#### 4.5.1. Analysis of Blank Solutions

We preliminary analyze release data from blank solutions. The donor compartment was loaded with 0.1 mL of furosemide solution 0.18 mg/mL or 0.18 mg/mL furosemide +5% *w*/*v* HP-β-CD. Therefore, $\delta_{d}=1$ mm, $M_{0}=18$ μg.

Diffusivity of furosemide in the solvent solution $D_{0}^{f}$ is preliminary estimated from Wilke–Chang’s relation

(39) $$D_{WC}\left[\frac{cm^{2}}{s}\right]=7.4\times 10^{-8}\frac{\sqrt{\Psi_{B}MW_{B}T)}}{\eta_{B}V_{LB}^{0.6}}$$

where B indicates the solvent (water, $\Psi_{B}$ = 2.26), and $V_{LB}$ = 317.6 is the LeBas drug molar volume of furosemide obtained from a group contribution approach, considering the contributions of individual atoms, functional groups, and cycles composing each molecule [27].

**Figure 7:**
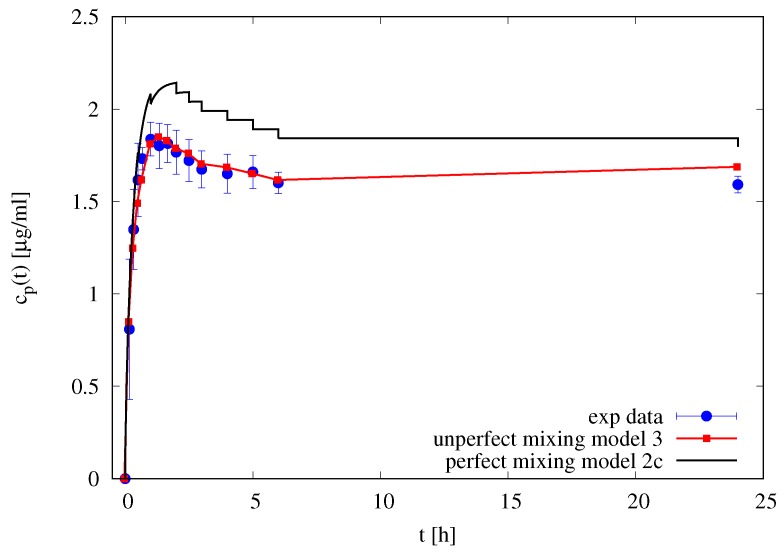
Differential release profile of furosemide blank solution. Filled circles represent experimental withdrawal concentrations $c_{p}\left(t_{i}\right)$. Continuous black line represents perfect mixing model predictions (Model IIc). The red line with square points represent the imperfect mixing model predictions (Model III).

Figure 7 shows the experimental differential release curve (withdrawal concentrations $c_{p}\left(t_{i}\right)$ at different time instants) and the two models adopted: the perfect mixing Model IIc and the imperfect mixing Model III with $D^{d}=D_{0}^{d}=D_{0}^{f}=5.78\times 10^{-10}$ m${}^{2}$/s and $D_{m}^{d}=0.5D_{0}^{d}$. It can be observed that the imperfect mixing model is capable to accurately predict the differential release curve, while the perfect mixing model (including the effect of withdrawals) significantly overestimate the withdrawal concentrations.

This preliminary observation supports our initial hypothesis that only the imperfect mixing model, when applied directly to differential release curve (and not to the integral release curve) led to a correct estimate of drug diffusivity values.

**Figure 8:**
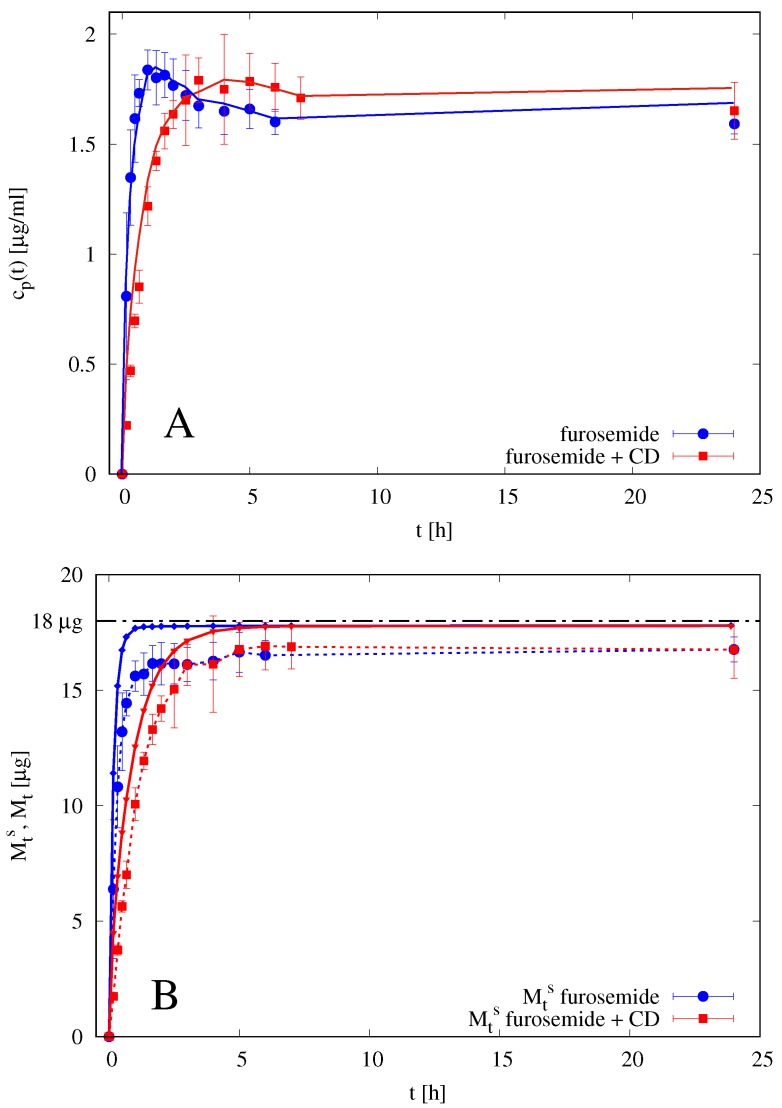
Differential (**A**) and integral release curves (**B**) for blank solution of furosemide with and without HP-β-CD. Continuous lines represent the imperfect mixing Model III predictions with $\delta_{d}=1$ mm, $\delta_{m}=45$μm, $D_{0}^{f}=5.78\times 10^{-10}$ m${}^{2}$/s, $D_{m}^{f}=0.5D_{0}^{f}$, $D_{0}^{f+CD}=1.73\times 10^{-10}$ m${}^{2}$/s and $D_{m}^{f+CD}=0.25D_{0}^{f+CD}$. (**A**) Differential release curves $c_{p}\left(t_{i}\right)$. (**B**) Integral release curves. $M_{t}^{s}$ dashed curves with points represent the integral release curves as evaluated from experimental data and Equation (20) by assuming a perfectly mixed accepting compartment $V_{res}=7.9$ mL. Continuous lines represent the integral release curves as evaluated from the imperfect mixing Model III. Horizontal dot-dashed line represent the amount of drug $M_{0}$ initially loaded in the donor compartment.

The imperfect mixing model is therefore applied to differential release data of 0.1 mL of furosemide solution 0.18 mg/mL furosemide +5% *w*/*v* HP-β-CD thus obtaining the following estimate for the diffusivity of the furosemide/HP-β-CD complex $D_{0}^{f+CD}=1.73\times 10^{-10}$ m${}^{2}$/s as obtained from the best fitting of data reported in Figure 8A with $D_{m}^{f+CD}=0.25D_{0}^{f+CD}$. The diffusivity of furosemide/HP-β-CD complex in the membrane has been assumed to be four times smaller than the diffusivity in the solvent solution given the large dimension of the complex and the membrane cutoff. The resulting diffusivity of furosemide/HP-β-CD complex $D_{0}^{f+CD}$ is significantly smaller than furosemide diffusivity $D_{0}^{f}$. A review of diffusivity values is reported in Table 2.

Figure 8B shows the integral release curves $M_{t}^{s}$ (dashed curves with point) as usually evaluated in the literature, i.e., from experimental data $c_{p}\left(t_{i}\right)$ and Equation (21), by assuming a perfectly mixed accepting compartment $V_{res}=7.9$ mL. It can be observed that the integral release curve $M_{t}^{s}$ significantly underestimates the total amount of drug asymptotically released, i.e., $M_{t}^{s}\left(\infty \right)<M_{0}$, while the imperfect mixing model (continuous lines) predicts $M_{t}\left(\infty \right)≃M_{0}$. The two integral curves $M_{t}$ and $M_{t}^{s}$ cannot coincide because they are based on different basic assumptions.

#### 4.5.2. Analysis of Release Data from Films

When the thin dry film is placed in the donor compartment of the Franz cell, it comes in contact with the solvent solution through the membrane and therefore swells and erodes. However, the eroded material is not washed away and removed, but it remains in the donor compartment. Therefore, we assume that the swollen film thickness Th is the thickness attained when the maximum swelling degree is reached, i.e.,

(40) $$Th=Th_{dried-film}+Th_{water-uptake}=Th_{dried-film}+\left(\frac{W^{max}}{W_{0}}\right)\frac{W_{0}}{\rho_{s}A}$$

where $W^{max}/W_{0}≃3.5$, $\rho_{s}$ is the solvent density and *A* is the cross-section area of the donor compartment. The resulting values are Th≃450 μm for films without HP-β-CD and Th≃580 μm for films with HP-β-CD.

Given that the time scales of swelling are extremely small (order of 1 min), we can assume that the film in a release experiment in the Franz cell is fully swollen since from the first time instants.

From the release curves of furosemide loaded in thin films (with and without HP-β-CD) and placed in the donor compartment of a Franz cell, we can estimate the effective diffusivity of furosemide $D_{G}^{f}$ and of furosemide/HP-β-CD complex $D_{G}^{f+CD}$ in the swollen gel.

**Figure 9:**
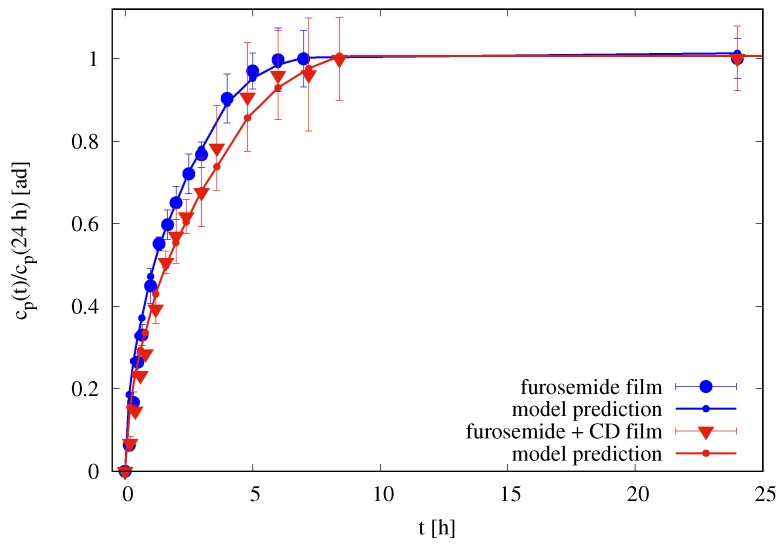
Rescaled differential release curves $c_{p}\left(t\right)/c_{p}$ (24 h) vs. *t* [h] for films in the Franz cell. Continuous lines (with small dots) represent imperfect mixing model III predictions with $\delta_{m}=45$μm, $\delta_{d}=450$μm for films without HP-β-CD, and $\delta_{d}=580$μm for films with HP-β-CD. Diffusivity values $D_{G}^{f}$, $D_{G}^{f+CD}$, $D_{m}^{f}$, and $D_{m}^{f+CD}$ are reported in Table 2.

The imperfect mixing model is therefore applied to differential release data of furosemide from thin films in Franz-cell with $\delta_{d}=Th$. Figure 9 shows the comparison between experimental differential data $c_{p}\left(t_{i}\right)$ for films with and without HP-β-CD and the model predictions with a best fit value of effective diffusivity of furosemide in the swollen gel $D_{G}^{f}=6.5\times 10^{-11}$ m${}^{2}$/s and a best fit value of effective diffusivity of furosemide/HP-β-CD complex $D_{G}^{f+CD}=5.4\times 10^{-11}$ m${}^{2}$/s. The effective diffusivities in the swollen gel are very close for both furosemide and furosemide/HP-β-CD complex, $D_{G}^{f}≃1.2\times D_{G}^{f+CD}$ and significantly smaller than diffusivities in the solvent solution $D_{0}^{f}$ and $D_{0}^{f+CD}$. Diffusivities in the membrane are the same as obtained from blank data, i.e., $D_{m}^{f}=0.5D_{0}^{f}$ for film without HP-β-CD and $D_{m}^{f+CD}=0.25D_{0}^{f+CD}$. A review of diffusivity values is reported in Table 2.

Estimated diffusivities are directly used in the transport models for drug release in the MFTD and in the USP II apparatus.

**Figure 10:**
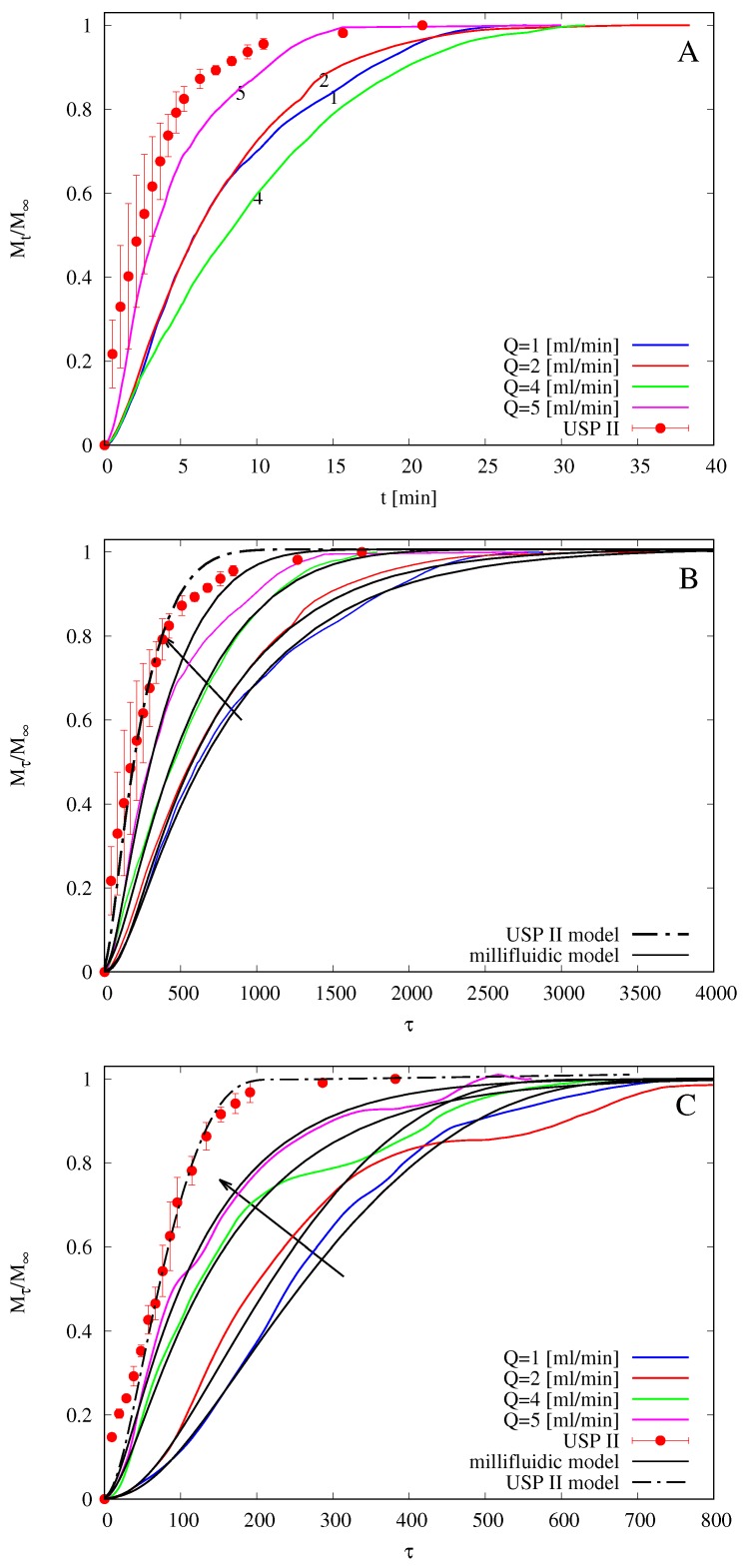
Drug releases at 37${}^{\circ }C$ from USP II apparatus (paddle, 50 rpm) and from MFTD for different flow rates Q=1,2,4,5 mL/min. (**A**) $M_{t}$ vs. *t*, films without HP-β-CD. (**B**) $M_{\tau }$ vs. $\tau =tD_{G}^{s}/L_{0}^{2}$, films without HP-β-CD. (**C**) $M_{\tau }$ vs. τ, films with HP-β-CD. Arrows indicate increasing values of *Q*.

It must be observed that release experiments from erodible films in the Franz cell cannot be used to estimate the actual release time scales in USP II or MFTD, because drug release in a Franz cell is unaffected by erosion (the eroded material remains in the donor compartment). For this reason, we observe that an almost complete release requires 10 h in the Franz cell and 10–15 min in USP II or MFTD (see Figure 10 in Section 4.6). Release data in the Franz cell have been exclusively used to estimate the drug effective diffusivities in the swollen gel.

### 4.6. Analysis of Release Kinetics in USP II and MFTD Apparatuses

In a recent work [14], drug release tests of commercially available melatonin strips obtained with the flow-through device were compared with those obtained using the official USP XXXVII basket (USP I) and paddle (USP II) apparatuses. The authors observed that, for flow rates comparable to salivary flow rates (*Q* = 2–4 mL/min), the MFTD shows much slower release profiles by approximately 10–15 min of delay with respect to the other two investigated methods. Additionally, in the present case, we observe that the official method (USP II) seems to significantly overestimate the release kinetics (and therefore to underestimate the time for complete drug release) when compared to the millifluidic device that mimics mouth physiological conditions thanks to the laminar tangential solvent flow, low flow rates, and low hold-up volume.

Figure 10A shows integral release curves $M_{t}/M_{\infty }$ vs. *t* (min) as obtained with the USP II apparatus and with the MFTD with flow rates Q=1,2,4,5 mL/min. No error bars for MFTD data (repeated in triplicate) are reported because of the large amount of data (output drug concentration is recorded every 2 s from the UV/Vis analyzer), but the maximum standard deviation is an order of 10%, i.e., $c_{s}\left(t_{i}\right)=\bar{c_{s}\left(t_{i}\right)}\left(1\pm 0.1\right)$, where $\bar{c_{s}\left(t_{i}\right)}$ represents the output concentration at time instant $t_{i}$, averaged over three repeated experiments.

It should be observed that release curves from the MFTD are extremely sensitive to initial film thickness $L_{0}$. The larger $L_{0}$, the larger the release time scales, for the same solvent flow rate. Release curves in the MFTD are also extremely sensitive to the solvent flow rate *Q*. The larger *Q* is, the smaller the mass-transfer resistance is at the gel-solvent interface and therefore the faster the release is. Actually, for very high flow rates (Q>12 mL/min), the dissolved drug is immediately swept away by the solvent flow, and diffusion through the polymeric matrix becomes the controlling step. At this point, any further increase in the flow rate does not improve drug release.

Release curves shown in Figure 10 seem not to follow the expected behavior (the larger *Q* is, the faster the release is) only because different experiments for different flow rates are characterized by significantly different average film thicknesses $L_{0}$. For example, $L_{0}≃70$ μm for Q=2, while $L_{0}≃90$ μm. For this reason, in order to make release data independent of the film thickness and to observe the expected behavior as a function of the flow rate *Q*, we choose, analogously to what has been done in Section 4.4 with swelling-erosion data, to represent release curves as a function of the dimensionless time $\tau =t/t_{ref}$, $t_{ref}$ being the characteristic swelling time $t_{ref}=L_{0}^{2}/D_{G}^{s}$.

Figure 10B shows the same integral release curves shown in Figure 10A but plotted as a function of the dimensionless time τ. We can readily observe that release data from USP II apparatus are significantly faster than that obtained in the MFTD and that release data from the MFTD follow the expected behavior as a function of *Q*. The same phenomenon is observed for release data from films including HP-β-CD, as shown in Figure 10C.

In Figure 10B,C, we also show integral release curves as obtained from the numerical solution of theoretical models for USP II and MFTD described in Section 3.3 and Section 3.4. All parameters entering the models, namely $D_{0}^{f}$, $D_{0}^{f+CD}$, $D_{G}^{f}$, and $D_{G}^{f+CD}$ are the same as estimated from release data in the Franz cell (reported in Table 2). The only parameter left as a best fitting parameter is the constant *C* [μm/s] entering the disentanglement rate equation (Equation (8)) in the swelling-erosion model, because it depends on the fluid dynamic conditions occurring in the dissolution apparatus.

**Figure 11:**
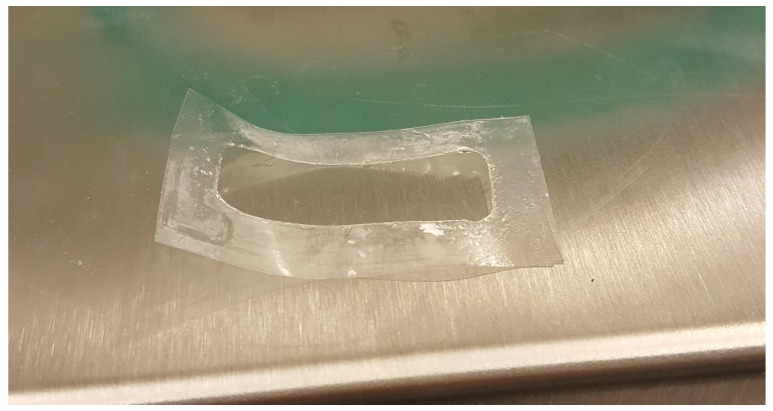
Detail of erosion effect on a film after complete release in the MFTD. The portion of the film exposed to the solvent is completely removed at the end of the experiment.

We expect that erosion, quantified by the disentanglement rate, is maximum in the USP II apparatus and that $R_{dis}$ increases with the flow rate *Q* in the MFTD, because the larger the flow rate is, the larger the shear stress at the gel-solvent interface is (see Figure 11 for a detail of erosion effect on a film after complete release in the MFTD). This is confirmed by the analysis of the disentanglement rate $R_{dis}$ estimated from a best fit of experimental release data and reported in Table 3. Erosion is also responsible for the “wavy” behavior of the MFTD release curves due to the possible detachment of small pieces of film that can affect UV/Vis detection of drug concentration.

The agreement between model prediction and experimental data is quite satisfactory for both films with and without HP-β-CD. In agreement with swelling-erosion experiments, drug release is faster for films with HP-β-CD due to larger erosion effects. For both films the release in USP II apparatus is at least twice as fast as that in MFTD at an intermediate salivary flow rate Q=2,3 mL/min.

## 5. Conclusions

In this work, hydroxypropylmethyl cellulose (HPMC) fast-dissolving thin films containing hydroxypropyl-β-CD are proposed as suitable formulations for furosemide oral drug delivery.

Three different apparatuses, namely the Franz cell, the millifluidic flow-through device, and the USP II paddle type dissolution apparatus, were used and the results compared.

In particular, we propose an original use of the Franz cell as a release apparatus for estimating the effective drug diffusion coefficient in the swollen film, especially when, as in the present case, OTFs exhibit rapid disintegration (strong erosion effects).

The millifluidic flow-through device, specifically designed to mimic mouth physiological conditions, allowed us to estimate drug release time scales at solvent flow rates comparable with salivary flow rates and to observe significant and expected differences with release time scales estimated with official methods, e.g., the USP II apparatus, actually designed for mimic the gastrointestinal tract.

Swelling-erosion data show that OTFs undergo a rapid swelling and erosion. Release data in MFTD and USP II show that OTFs exhibit fast release, enhanced by rapid dissolution.

In agreement with swelling-erosion experiments, furosemide release is faster for films including HP-β-CD due to larger erosion effects. For both films, with and without HP-β-CD, the release in USP II apparatus is at least twice faster than release in MFTD at an intermediate salivary flow rate Q=2,3 mL/min, thus confirming that official USP II apparatus tends to overestimate the release kinetics.

Mathematical models adopted allowed us to estimate all the diffusion coefficients required for an accurate description of drug release in MFTD and USP II.

The agreement between model predictions and experimental data is quite satisfactory for both films with and without HP-β-CD. The erosion effect, quantified by the disentanglement rate, is influenced by the fluid dynamic conditions characterizing the release apparatus. It is maximum in the USP II apparatus and increases with the flow rate *Q* in the MFTD.

Although mucoadhesion tests should be performed in order to verify if the residence time “in situ” of the formulation is comparable with that required for drug release, the analysis reported and the results obtained showed that HPMC-based thin films represent a valid drug delivery and fast release formulation.

## Figures and Tables

**Table 1:** Parameters entering the swelling-erosion model.

Film	$L_{0}$ (μm)	$\phi_{0}$	$\phi_{g}$	$\phi_{\mathrm{eq}}$	$D_{G}^{s}$ (m${}^{2}$/s)	*C* (μm/s)	$R_{\mathrm{dis}}$ (μm/s)
without HP-β-CD	87 ± 5	0.21	0.3	0.89	$7.95\times 10^{-9}$	67.72	1.87
with HP-β-CD	126 ± 5	0.12	0.18	0.85	$7.95\times 10^{-9}$	84.37	3.86

**Table 2:** Diffusivity values (m${}^{2}$/s) estimated from release data in Franz cell.

$D_{0}^{f}$	$D_{m}^{f}=0.5\;D_{0}^{f}$	$D_{0}^{f+\mathrm{CD}}$	$D_{m}^{f+\mathrm{CD}}=0.25\;D_{0}^{f+\mathrm{CD}}$	$D_{G}^{f}$	$D_{G}^{f+\mathrm{CD}}$
$5.78\times 10^{-10}$	$2.89\times 10^{-10}$	$1.73\times 10^{-10}$	$4.32\times 10^{-11}$	$6.5\times 10^{-11}$	$5.4\times 10^{-11}$

**Table 3:** Disentanglement rate (μm/s) for release experiments in USP II apparatus and in MFTD at different flow rates.

$R_{\mathrm{dis}}$ (μm/s)	1 (mL/min)	2 (mL/min)	4 (mL/min)	5 (mL/min)	USP II
without HP-β-CD	0.26	0.32	0.41	0.5	0.64
with HP-β-CD	0.38	0.47	0.61	0.74	0.96

## Supplementary Materials

The following are available at http://www.mdpi.com/1999-4923/10/4/222/s1, Figure S1: Flow curves of the film-forming solutions, Figure S2: Puncture strength (A) and elongation to break (B) of OTFs with and without HP-β-CD.
