# Supplementary material for: Experimental and Modeling Study of Drug Release from HPMC-Based Erodible Oral Thin Films
Source: Pharmaceutics. 2018 Nov 9;10(4):222. doi: 10.3390/pharmaceutics10040222 (PMC6320981; doi:10.3390/pharmaceutics10040222)
Supplement: Supplementary file 1 [file pharmaceutics-10-00222-s001.pdf]

pharmaceutics

Supplementary Materials: Experimental and Modeling Study of Drug Release From HPMC-Based Erodible Oral Thin Films

Alessandra Adrover, Gabriele Varani, Patrizia Paolicelli, Stefania Petralito, Laura Di Muzio, Maria Antonietta Casadei and Ingunn Tho

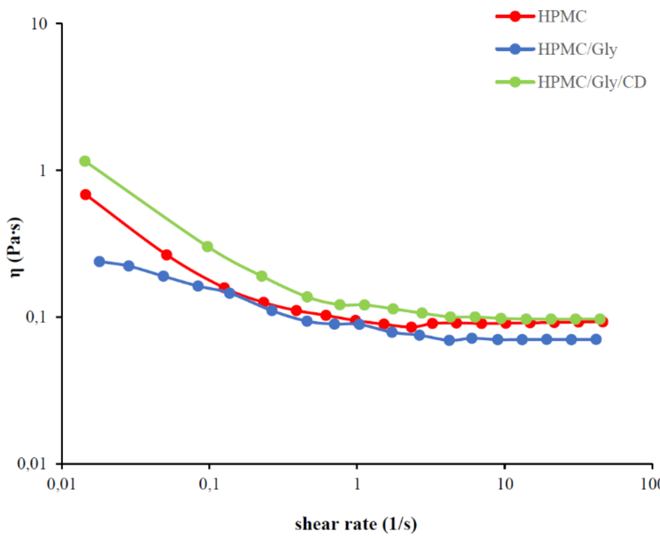

Figure S1. Flow curves of the film-forming solutions.

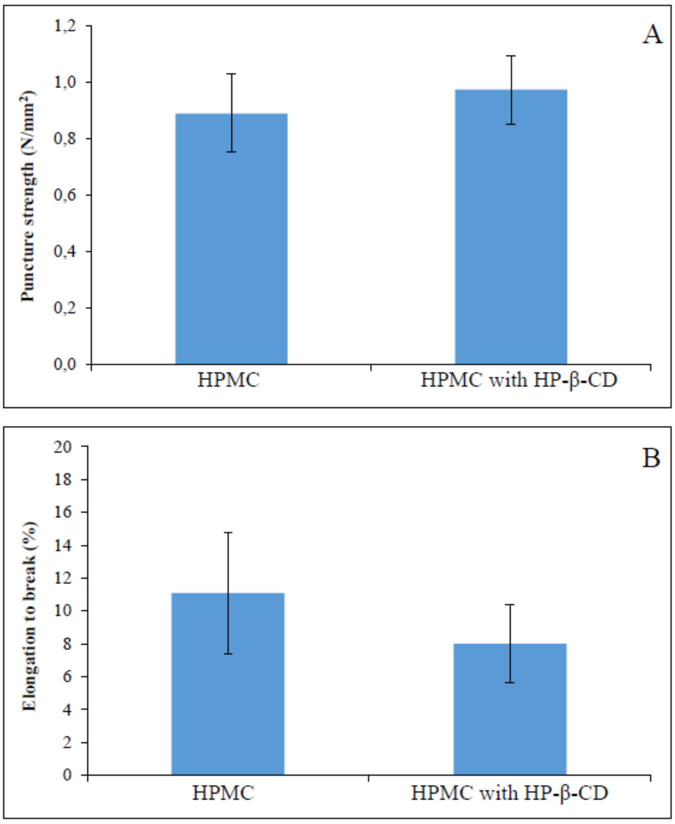

Figure S2. Puncture strength (A) and elongation to break (B) of OTFs with and without HP-β-CD.
